# Giant bilateral axillary artery aneurysms with left complete obstructive thrombus in intravenous immunoglobulin-sensitive Kawasaki disease: a case report

**DOI:** 10.1186/s12969-021-00643-w

**Published:** 2021-11-08

**Authors:** Chen Chu, Lan He, Yi-xiang Lin, Li-ping Xie, Fang Liu

**Affiliations:** Heart Center, Children’s Hospital of Fudan University, National Children’s Medical Center, 399 Wan Yuan Road, Shanghai, 201102 China

**Keywords:** Kawasaki disease, Systemic artery aneurysm, thrombus, Coronary artery aneurysm, Antithrombotic therapy

## Abstract

**Background:**

Kawasaki disease (KD) is a systemic vasculitis that predominantly affects medium-sized arteries. In addition to well-known coronary artery aneurysms (CAAs), peripheral systemic artery aneurysms (SAAs) have also been sporadically reported. In the literatures, SAAs occurred mainly in untreated, intravenous immunoglobin (IVIG)-resistant, or severe refractory KD, and thrombotic events in SAAs were rarely reported.

**Case presentation:**

A 10-month-old boy with a history of KD was referred to our hospital for suspected pseudoaneurysm of the axillary arteries. Four months prior to presentation, he had persistent fever, conjunctival congestion, and rash. On the 10th day of fever echocardiogram showed biliteral CAAs. He was then diagnosed with KD and given IVIG 2 g/kg and aspirin at a local hospital. His fever and symptoms soon subsided and he was discharged with low dose aspirin and dipyridamole. One month prior to presentation, his parents incidentally palpated swellings in his bilateral axillae. On admission, physical examination revealed a pulsatile swelling in his right axilla and a non-pulsatile swelling in the left with impalpable left brachial and radial pulses, cooler and less active left upper limb than the right one. While the pulses of other three limbs were normal. Ultrasound examination revealed giant bilateral axillary artery aneurysms (AAAs) with massive thrombus in the left. Angiography confirmed giant bilateral AAAs with left AAAs completely occluded and fine collateral vessels connecting to the distal brachial artery, in addition to giant bilateral multiple CAAs without stenoses. The patient was given intravenous prostaglandin for 10 days to allow for formation of collateral circulation, as well as aspirin, low molecular weight heparin (which was switched to warfarin before discharge) and metoprolol. At discharge, the temperature and movement of his left upper limb improved significantly. On follow-up at 7 months, his left upper limb further improved and was similar to the right with no occurrence of cardiovascular events. The images of CAAs and AAAs on echocardiogram and computerized tomography remained the same.

**Conclusions:**

This case highlights the importance of evaluating peripheral SAAs in KD patients with CAAs, even if their course of treatment appears smooth. For both large non-aortic SAAs and CAAs in KD patients, antithrombotic therapy is of utmost importance.

## Background

Kawasaki disease (KD) is a systemic vasculitis that predominantly affects medium-sized arteries. In addition to well-described coronary artery aneurysms (CAAs), it can also cause aneurysms of non-coronary systemic arteries [1]. The incidence of systemic artery aneurysms (SAAs) confirmed by angiography in untreated KD children has been reported to be approximately 2% [2], which was similar to a recent study conducted by our team in all the KD patients [3]. Previous literature [2–10], predominantly case reports, revealed that SAAs in KD patients were generally associated with coronary artery lesions, especially giant CAAs, and were more likely to occur in infants, in patients with untreated KD, intravenous immunoglobulin (IVIG) -resistant or severe refractory KD, which are all related to high risk of developing CAAs. In addition, thrombotic events related to SAAs are rarely reported. Here, we reported an IVIG-sensitive KD case who developed both giant CAAs and giant bilateral axillary artery aneurysms, accompanied with complete obstructive thrombus on the left.

## Case presentation

A 10-month-old boy was referred to our hospital because of bilateral axillary swellings. Four months prior to referral, the infant had persistent fever, followed by bilateral conjunctival congestion and a maculopapular rash on his limbs and trunk. Laboratory tests showed elevated leucocytes and C-reactive protein (CRP). His fever persisted after antibiotic therapy. On the 10th day of fever, echocardiographic examination at a local hospital showed dilation of bilateral coronary arteries (there were no documented diameters for each coronary artery). He was then diagnosed with KD and admitted to the local hospital immediately. Laboratory tests showed elevated CRP (59 mg/L), erythrocyte sedimentation rate (35 mm/h), leukocytes (16.9 × 10^9^/L) and platelets (658 × 10^9^/L), decreased hemoglobin (94 g/L), and normal hepatic enzymes, albumin and kidney tests. The infant was administrated a single dose (2 g/kg) of IVIG and aspirin (40 mg/kg/d q8h) on the day of admission. His fever subsided within 24 h after completing the IVIG infusion, with clinical symptoms and laboratory tests improved uneventfully. Before discharge, all the laboratory tests returned to normal except platelets (751 × 10^9^/L). Repeated echocardiography still showed bilateral CAAs. He was discharged after 9 days of hospitalization, with low dose aspirin and dipyridamole. The infant remained afebrile and was followed up at the local hospital due to restrictions related to the COVID-19 pandemic. Follow-up echocardiography showed no obvious changes of CAAs and he has been taking aspirin and dipyridamole. One month prior to referral, his parents incidentally felt swellings in his bilateral axillae, but without paying further attention. Three days before presentation to our hospital, an ultrasound done at the local hospital during his regular follow-up showed suspected axillary artery aneurysms or pseudoaneurysms. He was then immediately referred to our hospital for further evaluation.

On admission to our hospital, physical examination showed a pulsatile swelling in the right axilla, a non-pulsatile swelling in the left axilla, impalpable left brachial and radial pulses, lower skin temperature of the left upper limb than that of the right one, pale left palm, and less active movement of left upper limb than that of the right one. The pulses, skin temperature, and color of right upper limb and both lower extremities were all normal. The infant’s parents denied the family history of vasculitis or congenital arterial aneurysms, and his special medical history other than KD.

**Fig. 1:**
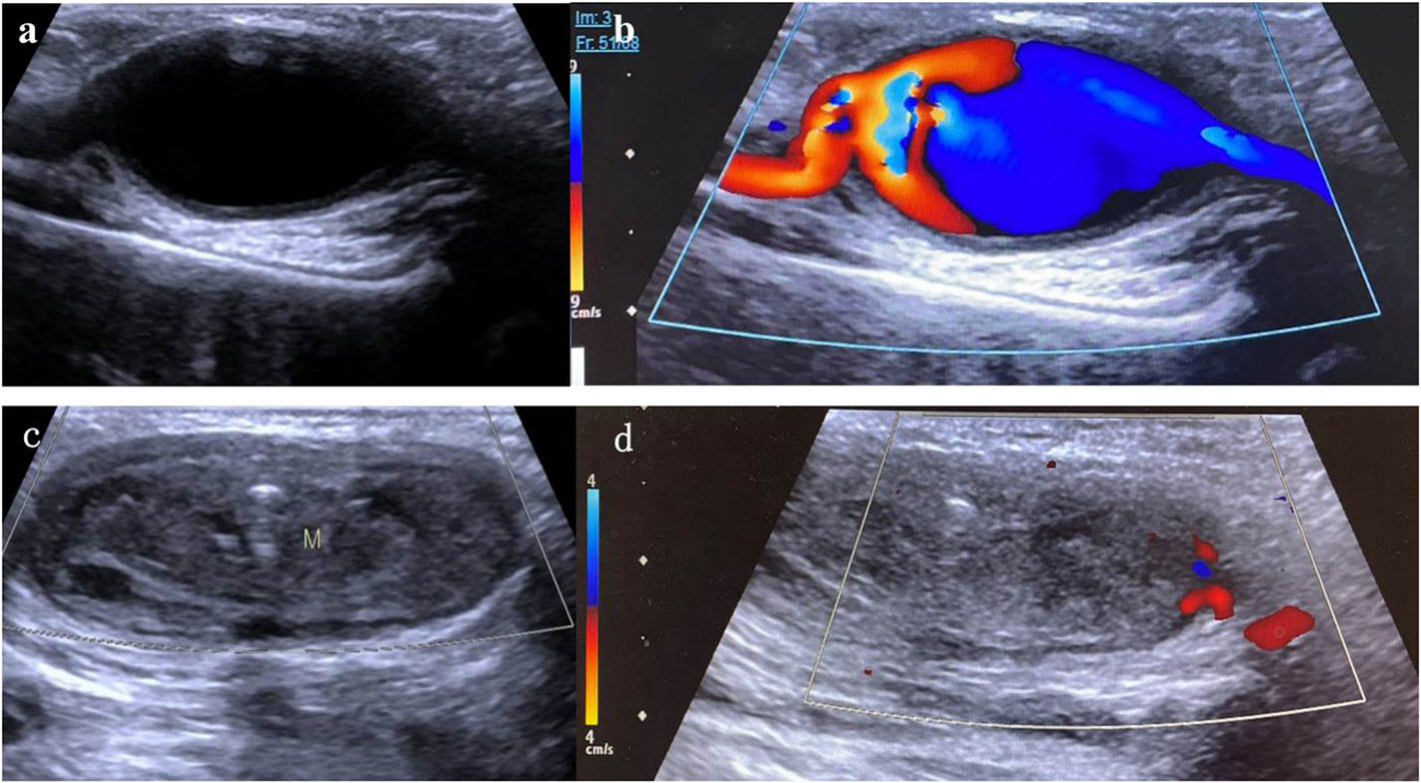
The ultrasound images of bilateral axillary artery aneurysm. **a**, **b** The right giant aneurysm (**a** without color Doppler; **b** with color Doppler) without thrombus, with the flow velocity of input and output site of the aneurysm 0.8 m/s and 0.6 m/s respectively; **c**, **d** The left giant aneurysm with massive thrombus occlusion (**c** without color Doppler; **d** with color Doppler) and sparse blood flow near the mass

After admission, the ultrasound examination showed a giant right axillary artery aneurysm (AAA) with a size of 26*12.7*19.5 mm and a wall thickness of 1.2 mm. The flow inside the aneurysm was patent with no stenosis and the diameters of the distal and proximal adjacent arteries were about 2.3–3.0 mm (Figs. 1a & b). In left axilla, a large heterogeneous mass (41.4*11.7*15.8 mm) without blood flow was detected, which suggested complete occlusion (Figs. 1c & d).

No abnormalities were found on echocardiography except giant multiple CAAs involving the left anterior descending artery (LAD) and right coronary artery (RCA). The internal diameters of the aneurysms in the LAD, the proximal RCA, and mid RCA were 7.48 mm (z score = + 18.65), 9.0 mm (z score = + 20.76) and 5.09 mm (z score = + 10.64), respectively. Investigations to rule out myocardial ischemia including 12-lead electrocardiogram, 24-h Holter monitor, 2-dimention and tissue Doppler echocardiography, and adenosine stressed nuclide myocardial perfusion imaging, were all normal.

**Fig. 2:**
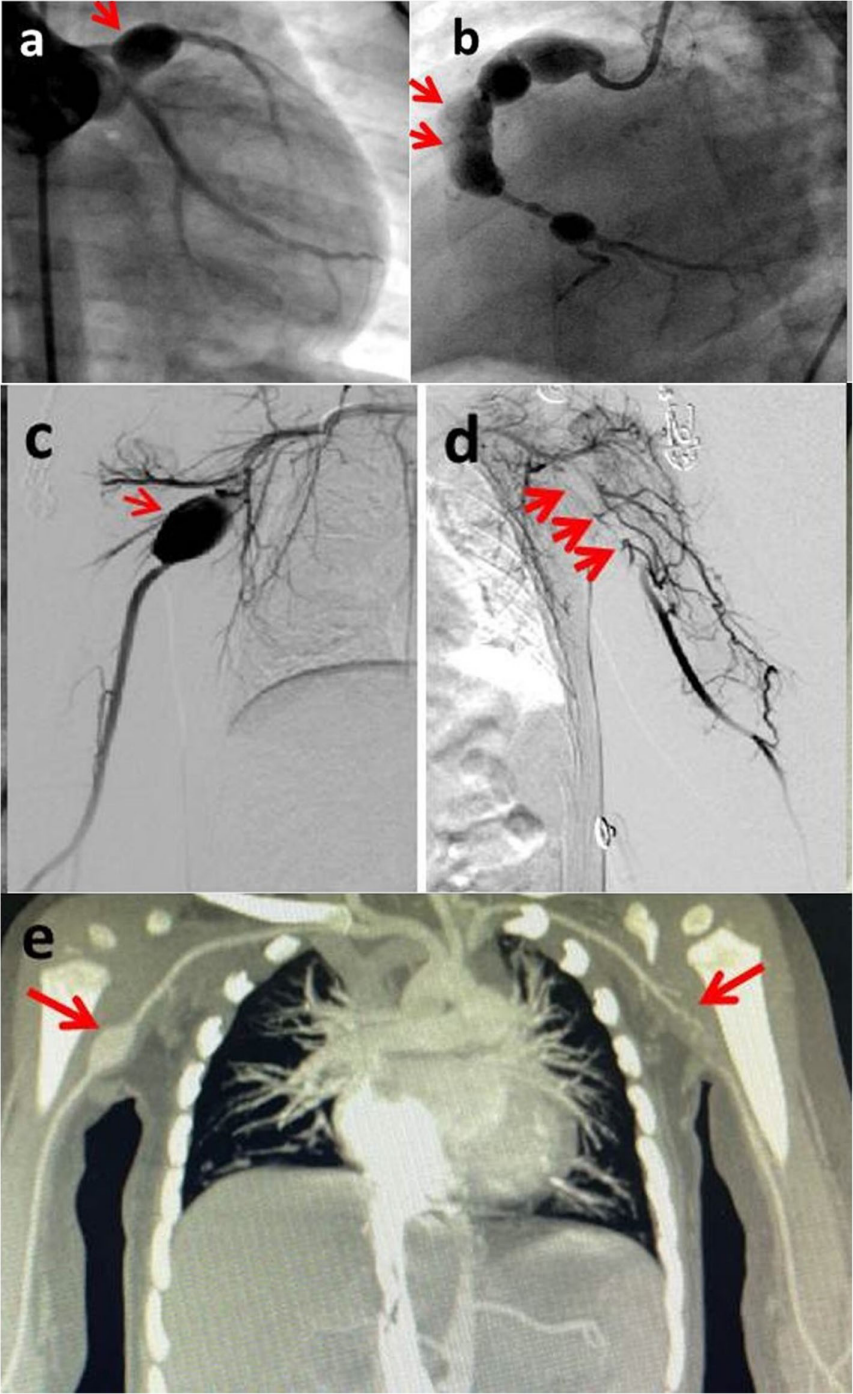
Coronary angiography shows: **a** A 6.0*8.5 mm aneurysm (arrow) at the bifurcation of LAD and circumflex branch. **b** Beaded aneurysms in the RCA from proximal branch to the bifurcation of the posterior descending coronary artery. An incomplete thrombus can be seen in the third aneurysm (arrows). Peripheral arteriography shows: **c** Right giant AAA (arrow) with normal blood flow. **d** Left giant AAA which has been completely occluded (arrows) with fine collateral vessels supplying blood to the distal brachial artery. **e** CT angiogram at 3-month follow-up shows more blood flow to the left brachial artery distal to the occluded AAA compared to the images 3 months prior (**d**)

Angiography was done for the patient. Coronary angiography showed giant multiple CAAs, including a 6.0*8.5 mm CAA at the bifurcation of the LAD and circumflex branch (Fig. 2a) and several beaded CAAs in RCA from proximal to the origin of the posterior descending coronary artery (the diameters of aneurysms were 9.0 mm, 7.6 mm, 6.8 mm, and 5.2 mm, respectively) (Fig. 2b). An incomplete thrombus could be seen in the third aneurysm of the RCA (Fig. 2b, arrows). Fortunately, the flow of the LCA and RCA was patent with no stenoses or occlusions. Left and right subclavian arteriography showed giant bilateral AAAs. The size of the right AAA was 15.5*25.0 mm, with no thrombus in the aneurysm (Fig. 2c). The blood flow throughout the right upper limb was normal. The size of left AAA was 16.5*45.0 mm, with complete thrombus inside the aneurysm. There were some fine collateral vessels connecting to the distal brachial artery, and the blood flow into the distal left upper limb was slow and sparse (Fig. 2d). No abnormalities of other peripheral arteries were found.

Laboratory tests including CRP, erythrocyte sedimentation rate, leukocytes, platelets, hemoglobin, serum amyloid protein, interleukin-6, and hepatic and kidney tests, were all normal when the peripheral SAAs were found.After completing evaluations and consulting with the vascular surgical team, we administrated intravenous prostaglandin for this baby to accelerate angiogenesis of the collateral vessels instead of surgical repair of the occluded aneurysm. At the same time, oral aspirin (5 mg/kg per day) and low molecular weight heparin (150u/kg/ dose q12h) were given to prevent thrombosis in the right AAA and CAAs. Metoprolol was used to reduce myocardial oxygen demand because of the giant CAAs. After 10 days of treatment, the patient’s skin temperature and movement of left upper limb had improved, and a weak pulse of left brachial artery could be palpable. Low molecular weight heparin was then switched to warfarin, with international normalized ratio (INR) monitored to be near 2.5.

As of writing this case report, the patient has been followed up for 7 months since discharge. During his stay at home, he was asymptomatic and grew well. Recent physical examination showed that skin temperature, movement, and pulses of his left upper limb further improved to a similar level to those of the right side. CT angiogram at 3-month follow-up confirmed that the blood flow of the brachial artery at the distal part of the occluded left axillary artery was significantly increased, and the right AAA had not changed (Fig. 2e). Repeat echocardiography showed the coronary arteries remained the same as before. Electrocardiogram was normal.

## Discussion

In addition to CAAs commonly seen in KD, non-coronary systemic artery aneurysms can also be a sequela of KD. In previous reports on SAAs in KD [3–5, 7, 8, 10], most of SAAs occured in infants, and in patients with untreated KD, IVIG-resistant KD or severe refractory KD, which are all at a higher risk of developing CAAs. In this case, although there was somewhat of a delay in the diagnosis and treatment of the patient, on the 10th day of onset, he responded to one dose of IVIG. As his CAAs occurred before treatment, it might be possible that his bilateral axillary aneurysms were already present at the same time. However, SAAs were not evaluated simultaneously, indicating a lack of attention to SAAs by the clinicians.

In addition to vasculitis, peripheral aneurysms in children can also be idiopathic, traumatic, infectious, or of other causes. If these aneurysms are large or cause symptoms, surgery is recommended considering the risk of rupture [11]. However, there is no consensus on the treatment of SAAs in children with KD. Previous studies on the prognosis of SAAs in KD showed that 80% of SAAs could regress to normal within 6 months, and a small number of aneurysms could be stenotic, but rupture had never been reported [2, 3, 12]. In addition, the SAAs of KD mainly occurred in medium-sized arteries rather than the aorta. Therefore, there is no high risk of rupture even for large SAAs. Similarly, ruptures of large CAAs secondary to KD have rarely been reported and usually occur in the acute phase [1]. Actually, in previous cases, surgical or interventional treatment of aneurysms has been reported in only a few cases. Considering the risk of rupture, two KD patients separately complicated with giant aortic arch aneurysms and thoracoabdominal aortic aneurysms underwent aneurysm resection and aortic reconstruction with artificial conduits [13, 14]. Another two cases of KD separately with hepatic and intercostal aneurysms were treated with percutaneous coil embolization, in order to maintain local artery circulation [15, 16]. Therefore, for large non-aortic aneurysms secondary to KD, there is no need to pay much attention to the risk of rupture.

Although the treatment of SAAs was not mentioned in the guidelines [1, 17], in view of the pathological characteristics of KD vasculitis, the main treatment principle of SAAs should be thromboprophylaxis, which is the same as that of CAAs. For CAAs in KD patients, thromboprophylaxis according to the risk-stratification of coronary artery abnormalities have been clearly recommended [1, 17]. Antiplatelet drugs (aspirin or clopidogrel instead) and anticoagulants (warfarin) are recommended in KD patients with large and giant CAAs. Therefore, KD patients with large SAAs should also need an anticoagulant regimen of both aspirin and warfarin just like those with large CAAs.

In this case, giant axillary aneurysms and CAAs were not detected in time at the local hospital, and only aspirin and dipyridamole were given for thromboprophylaxis, resulting in complete occlusion of the left AAA and associated ischemic symptoms. Considering the small diameters of the infant’s blood vessels and the formation of collateral vessels, the vascular surgeon suggested vasodilators be given in order to allow further developing of collateral circulation, instead of intervening with surgical bypass. At the same time, adequate antithrombotic treatment was given to maintain patency of the giant right AAA and multiple large CAAs. The right AAA was located in the axilla, which is relatively hidden, so the risk of rupture caused by external force is very small. However, his parents have been advised not to hold the baby with their hands in these areas. Follow-up examinations confirmed that the status of the right large AAA was stable, and the distal blood supply of the left upper limb had further improved.

## Conclusion

This case highlights the importance of evaluating peripheral SAAs in KD patients who are treated relatively late or have initial coronary artery involvement, even if patients are sensitive to IVIG treatment. For large non-aortic SAAs in KD patients, the risk of thrombosis should be given precedence over concerns of aneurysm rupture, and antithrombotic therapy is of utmost importance.

## Data Availability

Not applicable.

## Abbreviations

KD: Kawasaki disease
CAAs: Coronary artery aneurysms
IVIG: Intravenous immunoglobulin
SAAs: Systemic artery aneurysms
AAAs: Axillary artery aneurysms
LAD: Left anterior descending artery
RCA: Right coronary artery
CRP: C-reactive protein
